# Clinicopathological and prognostic significance of EGFR, VEGF, and HER2 expression in cholangiocarcinoma

**DOI:** 10.1038/sj.bjc.6604129

**Published:** 2007-12-18

**Authors:** D Yoshikawa, H Ojima, M Iwasaki, N Hiraoka, T Kosuge, S Kasai, S Hirohashi, T Shibata

**Affiliations:** 1 Cancer Genomics Project, National Cancer Center Research Institute, Tokyo, Japan; 2 Division of Gastroenterological and General Surgery, Department of Surgery, Asahikawa Medical College, Asahikawa, Japan; 3 Pathology Division, National Cancer Center Research Institute, Tokyo, Japan; 4 Epidemiology and Prevention Division, Research Center for Cancer Prevention and Screening, National Cancer Center, Tokyo, Japan; 5 Hepato-Biliary and Pancreatic Surgery Division, National Cancer Center Hospital, Tokyo, Japan

**Keywords:** cholangiocarcinoma, epidermal growth factor receptor, vascular endothelial growth factor, human epidermal growth factor receptor 2, immunohistochemistry, prognosis

## Abstract

Epidermal growth factor receptor (EGFR), vascular endothelial growth factor (VEGF), and human epidermal growth factor receptor 2 (HER2) have been considered as potential therapeutic targets in cholangiocarcinoma, but no studies have yet clarified the clinicopathological or prognostic significance of these molecules. Immunohistochemical expression of these molecules was assessed retrospectively in 236 cases of cholangiocarcinoma, as well as associations between the expression of these molecules and clinicopathological factors or clinical outcome. The proportions of positive cases for EGFR, VEGF, and HER2 overexpression were 27.4, 53.8, and 0.9% in intrahepatic cholangiocarcinoma (IHCC), and 19.2, 59.2, and 8.5% in extrahepatic cholangiocarcinoma (EHCC), respectively. Clinicopathologically, EGFR overexpression was associated with macroscopic type (*P*=0.0120), lymph node metastasis (*P*=0.0006), tumour stage (*P*=0.0424), lymphatic vessel invasion (*P*=0.0371), and perineural invasion (*P*=0.0459) in EHCC, and VEGF overexpression with intrahepatic metastasis (*P*=0.0224) in IHCC. Multivariate analysis showed that EGFR expression was a significant prognostic factor (hazard ratio (HR), 2.67; 95% confidence interval (CI), 1.52–4.69; *P*=0.0006) and also a risk factor for tumour recurrence (HR, 1.89; 95% CI, 1.05–3.39, *P*=0.0335) in IHCC. These results suggest that EGFR expression is associated with tumour progression and VEGF expression may be involved in haematogenic metastasis in cholangiocarcinoma.

Cholangiocarcinoma arises from the ductal epithelium of the bile duct tree and is classified anatomically into intrahepatic cholangiocarcinoma (IHCC) and extrahepatic cholangiocarcinoma (EHCC). The incidence and mortality rates of cholangiocarcinoma, especially those of IHCC, are increasing worldwide (Khan *et al*, 2005). Complete resection is the only way to cure the disease at present. Moreover, because cholangiocarcinoma is difficult to diagnose at an early stage and extends diffusely, most patients have unresectable disease at clinical presentation, and prognosis is very poor (5-year survival is 0–40% even in resected cases) (Khan *et al*, 2005; Sirica, 2005). Therefore, novel effective therapeutic strategies are urgently required to improve the prognosis. Among potential therapeutic targets, several studies have revealed overexpression of epidermal growth factor receptor (EGFR) or human epidermal growth factor receptor 2 (HER2) protein, amplification, and mutation of these genes (Ito *et al*, 2001; Aishima *et al*, 2002; Ukita *et al*, 2002; Altimari *et al*, 2003; Gwak *et al*, 2005; Nakazawa *et al*, 2005; Leone *et al*, 2006) as well as overexpression of vascular endothelial growth factor (VEGF) protein (Hida *et al*, 1999; Tang *et al*, 2006) in cholangiocarcinoma.

Epidermal growth factor receptor and HER2 are members of the ErbB receptor tyrosine kinase family. Binding of ligands, such as epidermal growth factor and transforming growth factor alpha (TGF*α*), to their extracellular ligand-binding domain initiates intracellular signalling cascades, leading to progression, proliferation, migration, and survival of cancer cells (Olayioye *et al*, 2000; Yarden and Sliwkowski, 2001). Vascular endothelial growth factor plays a key role in tumour-associated neo-angiogenesis, which contributes to providing a tumour with oxygen, nutrition, and a route for metastasis. It binds to VEGFR (vascular endothelial growth factor receptor), and leads to survival, proliferation, and migration of endothelial cell (Tabernero, 2007). Expression of these molecules has been reported to have prognostic significance in several cancers (Gusterson *et al*, 1992; Han *et al*, 2001; Nicholson *et al*, 2001; Des Guetz *et al*, 2006; Mohammed *et al*, 2007). Recently, agents targeted at these molecules have been used clinically, such as trastuzumab in breast cancer (Gonzalez Angulo *et al*, 2006), gefitinib, and erlotinib in non-small cell lung cancer, and bevacizumab in colorectal cancer (Tabernero, 2007). In cholangiocarcinoma, a phase II study of erlotinib (Philip *et al*, 2006) and some case reports of combined chemotherapy including cetuximab (Sprinzl *et al*, 2006; Huang *et al*, 2007) have been reported.

However, no previous studies have clarified associations between the expression of these molecules and clinicopathological factors or prognosis in patients with cholangiocarcinoma. To elucidate the biological significance and potential of these molecules as therapeutic targets, we investigated EGFR/VEGF/HER2 expression and attempted to elucidate their associations with various clinical features as well as patient survival in 236 cases of cholangiocarcinomas.

## MATERIALS AND METHODS

### Patients

A total of 236 patients with cholangiocarcinoma (male 160; female 76) who had undergone tumour resection and been diagnosed histologically as having adenocarcinoma of the bile duct at the National Cancer Center Hospital, Tokyo, between January 1991 and August 2004, were enrolled in the present study. Median patient age and follow-up period were 65 years and 875 days, and median tumour sizes of IHCC and EHCC were 4.8 and 3.0 cm, respectively. Detailed characteristics of patient with IHCC and EHCC are presented in Table 1, Table 2. All patients were followed for more than 100 days. Follow-up examination was performed using computed tomography, abdominal ultrasonography, and measurement of the serum carcinoembryonic antigen and carbohydrate antigen 19–9 (CA19-9) levels every 3–6 months. Recurrence was diagnosed by clinical, radiological, or pathological methods, but mainly by radiological evaluation including computed tomography and ultrasonography. Clinical and pathological profiles were obtained from the database of hepatobiliary tumours based on the medical records of the patients. This study was approved by the Ethics Committee of the National Cancer Center, Tokyo, Japan, and written informed consent was obtained from all patients.

All cases were anatomically classified into two groups: IHCC and EHCC. Tumours arising from the bilateral hepatic duct or distal common bile duct were classified as EHCC. The numbers of IHCC and EHCC cases were 106 and 130, respectively.

### Histological assessment

Tumour staging and histological classification were assessed according to *TNM Classification of Malignant Tumours* (Sobin and Wittekind, 2002) defined by the International Union Against Cancer (UICC) and the *World Health Organization Histological Classification of Tumours* (Hamilton and Altonen, 2000). Macroscopic types of IHCC were defined with reference to *General Rules for the Clinical and Pathological Study of Primary Liver Cancer* (Liver Cancer Study Group of Japan, 2003): (1) the mass-forming type (MF), which develops an apparent tumour in the liver; (2) the periductal infiltrating type (PI), which spreads along the bile duct; (3) the intraductal growth type (IG), which is confined within the bile duct, and divided into two groups: the mass-forming group (MF and MF mixed with PI or IG) and the non-mass forming group (PI and/or IG). Macroscopic types of EHCC were divided into polypoid type and non-polypoid type (including nodular, scirrhous constricting, and infiltrating types). Other clinicopathological factors were categorised into groups that are presented in Table 1 (IHCC) and Table 2 (EHCC). Because the classifications and clinicopathological factors used in IHCC and EHCC are different, statistical analyses were performed separately.

### Immunohistochemistry

Immunohistochemistry (IHC) for EGFR, VEGF, and HER2 was performed using a polymer-based method (Envision™+Dual Link System-HRP (Dako, DK-2600 Glostrup, Denmark)). Sources and dilutions of primary antibodies were as follows: anti-EGFR (mouse monoclonal, clone 31G7; Zymed, South San Francisco, CA, USA; 1 : 100), anti-VEGF (rabbit polyclonal; Zymed; 1 : 50), and anti-HER2 (rabbit polyclonal; Dako; 1 : 300).

Formalin-fixed, paraffin-embedded serial tissue sections (4 *μ*m) were placed on silane-coated slides for IHC. Sections cut through the maximum tumour diameter were selected for IHC evaluation. The sections were deparaffinised and rehydrated in xylene and grade-diluted ethanol (50–100%), and submerged for 20 min in 0.3% hydrogen peroxide with absolute methanol to block endogenous peroxidase activity. Antigen retrieval for EGFR, VEGF, and HER2 was carried out by adding Digest-all™3 pepsin solution (Zymed) at 37°C for 10 min for EGFR, near boiling in 0.01 M citrate buffer (pH 6.0) for 15 min for VEGF, and heating in 0.01 M citrate buffer at 121°C for 10 min by pressure cooker for HER2. After protein blocking, the sections were incubated with each primary antibody at room temperature for 1 h, followed by incubation with Envision+ Dual Link reagent at room temperature for 30 min, and visualised using 3,3′-diaminobenzidine tetrahydrochloride as a chromogen. Finally, the sections were counterstained with haematoxylin. Sections were gently rinsed in phosphate-buffered saline between the incubation steps.

### Evaluation of immunohistochemistry

All sections were evaluated by DY, HO, and TS without the knowledge of any clinical or pathological information, and cases for which consensus could not be reached were discussed to decide the evaluation. Based on the Herceptest™ (Dako) criteria, intensities of both EGFR and HER2 were defined as follows: 0, no membrane staining or membrane staining in ⩽10% cancer cells; 1+, faint and partial membrane staining in >10% cancer cells; 2+, moderate and complete membrane staining in >10% cancer cells; 3+, strong and complete membrane staining in >10% cancer cells. Intensities of VEGF were defined as follows: 0, no cytoplasmic staining or cytoplasmic staining in ⩽30% cancer cells; 1+, faint cytoplasmic staining, equivalent to the intensity of normal bile duct epithelium within the same section, in >30% cancer cells; 2+, moderate cytoplasmic staining in >30% cancer cells; 3+, strong cytoplasmic staining in >30% cancer cells. For cases showing mixed intensity, the predominant intensity was selected as the final IHC score. A final IHC score of 2+ or 3+ was defined as positive for expression of each protein.

### Statistical analysis

Associations between results of IHC and clinicopathological factors were assessed by χ^2^ test. Cumulative survival rates and survival curves were calculated by the Kaplan–Meier method, and log-rank test was performed for the comparison of survival curves. Cox's proportional hazard model was performed to estimate hazard ratio (HR) and 95% confidence interval (CI) of each outcome (death and recurrence). Multivariate analyses were performed using the factors identified to be risk factors for each outcome by univariate analyses, without UICC pT and UICC Stage, which are composed of other factors. All *P*-values reported are two-sided, and significance level was set at *P*<0.05. All statistical analyses were performed with the Statview 5.0 statistical software package (Abacus Concepts, Berkeley, CA, USA).

## RESULTS

### Expression of EGFR, VEGF, and HER2 protein in cholangiocarcinoma

**Figure 1:**
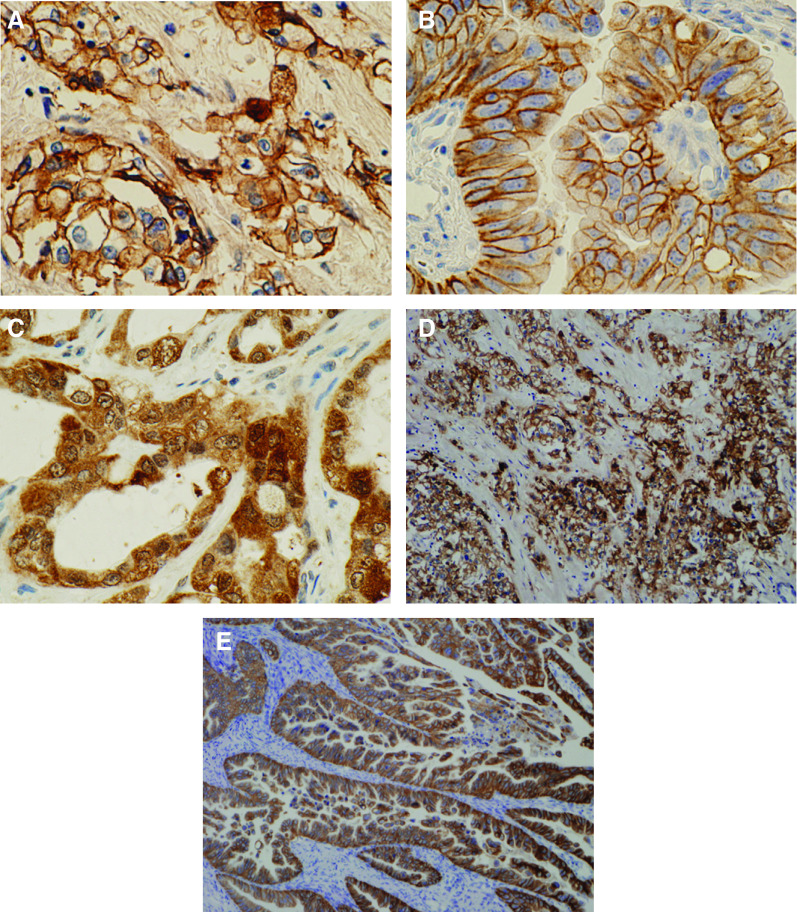
Representative immunohistochemical staining of (**A**) EGFR, (**B**) HER2, and (**C**) VEGF in cholangiocarcinoma (× 400 magnification). (**D**) Epidermal growth factor receptor tends to be expressed in the poorly differentiated component (× 100 magnification). (**E**) Human epidermal growth factor receptor 2 is preferentially expressed in more differentiated areas such as the glandular or papillary component (**×** 100 magnification).

Representative cases of positive staining for each protein are shown in Figure 1 (A, EGFR; B, HER2; C, VEGF). Epidermal growth factor receptor, VEGF, and HER2 were expressed in 29 (27.4), 57 (53.8), and 1 (0.9%) of the 106 IHCCs, respectively, and in 25 (19.2), 77 (59.2), and 11 (8.5%) of the 130 EHCCs, respectively. Microscopically, EGFR was mostly overexpressed in the moderately and/or poorly differentiated component, which is characterised by infiltration (52 of 54 EGFR-positive cases, Figure 1D), whereas only two cases showed EGFR overexpression in the well-differentiated component. In contrast, HER2 was preferentially expressed in the well-differentiated component. In 6 of 12 HER2-positive cases, HER2 was expressed only in well-differentiated component (Figure 1E), and 5 progressive cases showed positive HER2 staining in both the well and moderately and/or poorly differentiated components and 1 case only in moderately differentiated component. There was no association between VEGF expression and histological features.

### Associations between EGFR, VEGF, and HER2 expression and clinocopathological factors

Statistical analyses of HER2 were performed only in EHCC cases because of the small number of HER2-positive cases in IHCC. In IHCC, VEGF expression was significantly associated with intrahepatic metastasis (*P*=0.0224). There was no significant association between EGFR expression and any clinicopathological factors.

In EHCC, EGFR expression was significantly associated with macroscopic type (0% in the polypoid type, 24.0% in the non-polypoid type; *P*=0.0120), lymph node metastasis (*P*=0.0006), UICC Stage (*P*=0.0424), lymphatic vessels invasion (*P*=0.0371), and perineural invasion (*P*=0.0459). Human epidermal growth factor receptor 2 expression was significantly associated with macroscopic type (23.8% in the polypoid type, 5.8% in the non-polypoid type; *P*=0.0078), histological classification (25% in papillary adenocarcinoma, 9.7% in well differentiated adenocarcinoma, 3.2% in moderately differentiated adenocarcinoma, 5.9% in poorly differentiated adenocarcinoma; *P*=0.0237), and invasion to other organs (3.9% in invasive cases, 15.1% in non-invasive cases; *P*=0.0242). VEGF expression was not significantly associated with any factors in EHCC.

Detailed results of associations between EGFR/VEGF/HER2 expression and clinicopathological factors are shown in Supplementary information 1 (IHCC) and Supplementary information 2 (EHCC).

### Univariate and multivariate analyses regarding overall survival and tumour recurrence in cholangiocarcinoma

The number of dead and the median survival time were 70 cases and 724 days in IHCCs, and 76 cases and 1197 days in EHCCs, respectively. The number of recurrence and the median recurrence time were 64 cases and 522 days in IHCCs, and 78 cases and 960 days in EHCCs, respectively.

**Figure 2:**
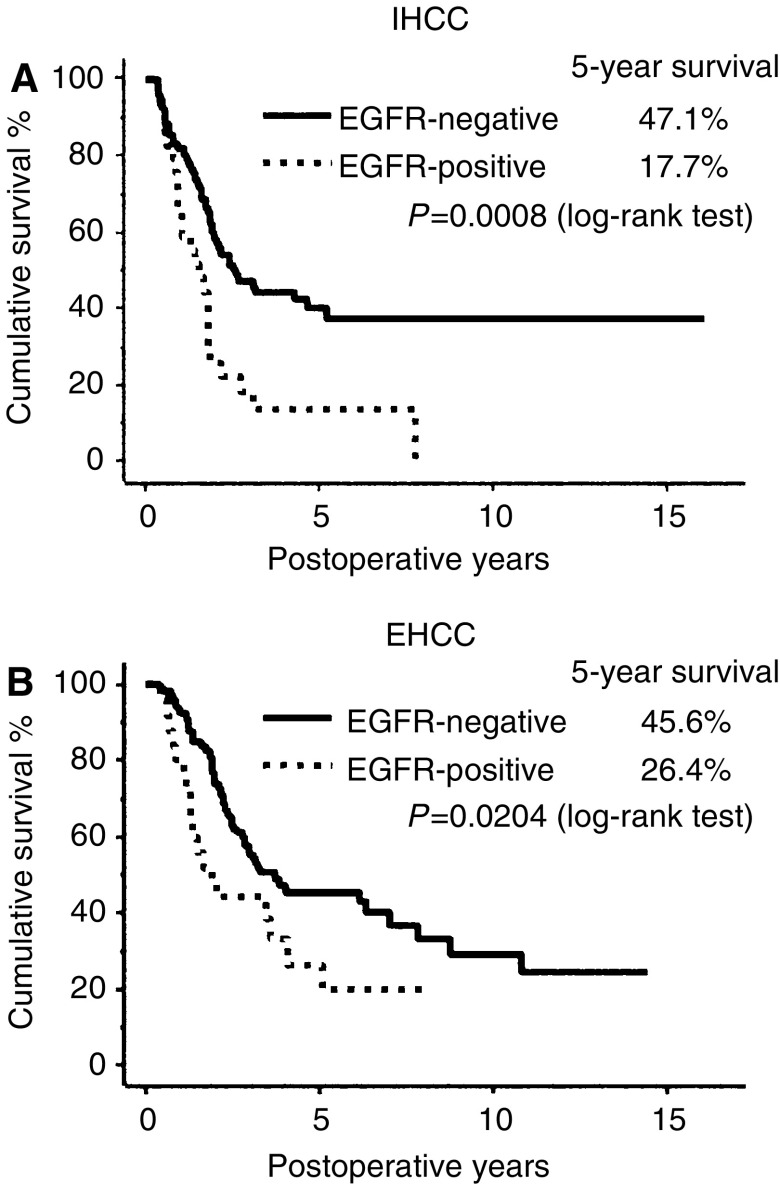
Survival curves stratified by EGFR expression in (**A**) IHCC and (**B**) EHCC (Kaplan–Meier method). The outcome of EGFR-positive cases was significantly worse than that of EGFR-negative cases in both IHCC (*P*=0.0008) and EHCC (*P*=0.0204) (by log-rank test).

Overall 5-year cumulative survival for patients with IHCC and EHCC was 33.0 and 41.6%, respectively, and no significant difference was identified between the groups (*P*=0.0599). The survival curves stratified by EGFR expression status are shown as Figure 2. Five-year survival for patients with EGFR-positive and EGFR-negative tumours was 17.7 and 47.1% for IHCC, and 26.4 and 45.6% for EHCC, respectively. There was a significant difference between EGFR-positive and -negative cases for both IHCC (*P*=0.0008) and EHCC (*P*=0.0204).

The results of multivariate analyses following univariate analyses regarding overall survival and tumour recurrence are shown in Table 3 (IHCC) and Table 4 (EHCC).

In IHCC, 13 factors including EGFR expression were identified as significantly prognostic by univariate analysis. Multivariate analysis revealed that EGFR expression was an independent prognostic factor (HR, 2.67; 95% CI, 1.52–4.69; *P*=0.0006), along with mass-forming macroscopic group (HR, 2.96; 95% CI, 1.06–8.31; *P*=0.0390), intrahepatic metastasis (HR, 2.91; 95% CI, 1.60–5.29; *P*=0.0005), and lymph node metastasis (HR, 1.96; 95% CI, 1.04–3.69; *P*=0.0375). In EHCC, 14 factors including EGFR expression were identified as significantly prognostic by univariate analysis. Multivariate analysis revealed that lymph node metastasis (HR, 2.03; 95% CI, 1.16–3.55; *P*=0.0133) and a histological classification of moderately differentiated adenocarcinoma (HR for papillary adenocarcinoma, 4.23; 95% CI, 1.08–16.50; *P*=0.0380) and poorly differentiated adenocarcinoma (HR for papillary adenocarcinoma, 13.22; 95% CI, 3.10–56.45; *P*=0.0005) were significant prognostic factors.

Multivariate analysis following univariate analysis for risk factors of tumour recurrence revealed that EGFR expression in IHCC was a significant risk factor of tumour recurrence (HR, 1.89; 95% CI, 1.05–3.39; *P*=0.0335), along with intrahepatic metastasis (HR, 2.36; 95% CI, 1.31–4.25; *P*=0.0044), lymph node metastasis (HR, 2.24; 95% CI, 1.19–4.22; *P*=0.0126), and venous invasion (HR, 6.74; 95% CI, 1.31–34.73; *P*=0.0225), whereas, in EHCC, lymph node metastasis (HR, 1.75; 95% CI, 1.03–2.98; *P*=0.0394) and dissected periductal structures margin (HR, 1.81; 95% CI, 1.03–3.16; *P*=0.0383) were independent risk factors of tumour recurrence, but EGFR expression was not associated with tumour recurrence even in univariate analysis.

## DISCUSSION

This study, analysing EGFR/VEGF/HER2 expression in the largest cohort of cholangiocarcinoma reported so far, showed for the first time that EGFR expression in IHCC is significantly associated with poor prognosis. In addition, our study confirmed previously reported prognostic factors in cholangiocarcinoma, such as macroscopic type, intrahepatic metastasis, lymph node metastasis, and histological classification (Yamamoto *et al*, 1998; Ohtsuka *et al*, 2002; Morimoto *et al*, 2003; DeOliveira *et al*, 2007). Expression of EGFR or HER2 is known to be a prognostic factor in some cancers (Gusterson *et al*, 1992; Nicholson *et al*, 2001), but no previous study has clarified the influence of these molecules on prognosis in cholangiocarcinoma (Ito *et al*, 2001; Altimari *et al*, 2003; Nakazawa *et al*, 2005), probably because cholangiocarcinoma is a relatively rare cancer and collection of a large cohort is difficult. Indeed, most previous studies were performed on the basis of only 50 cases at most. Although it is unclear why EGFR expression in IHCC is an independent prognostic factor, it may be associated with frequent relapse of cancer because EGFR expression is also a risk factor for tumour recurrence.

In contrast to IHCC, EGFR expression was not an independent prognostic factor in EHCC, but was associated with clinical features that may represent tumour progression and invasion, such as lymph node metastasis and apparent stromal invasion in EHCC. Because cancer tissue tends to be heterogeneous, histological diagnosis is generally decided on the basis of the degree of differentiation that predominates. In order to elucidate the biological significance of each protein, we microscopically examined positive cases in detail and compared their expression with histological components, and found that EGFR tended to be expressed in the poorly differentiated component, which is characterised by infiltration in both IHCC and EHCC. Similar results have been reported in bladder cancer (Neal *et al*, 1985), oesophageal adenocarcinoma (Wilkinson *et al*, 2004), and IHCC (Ito *et al*, 2001), although the studies were based on small cohorts. These findings indicate that EGFR expression may be a relatively late event in the development of cholangiocarcinoma and associated with invasion and progression. Because it has been previously reported that poor differentiation is associated with unfavourable outcome in other cancers (Sohn *et al*, 2000; Hassan *et al*, 2005), the association between EGFR expression and poor differentiation may also be a reason that EGFR expression is a prognostic factor.

Though the prognostic factors were different between IHCC and EHCC, it may be due to the difference of anatomical character, which extrahepatic bile duct is near from other organs and is not surrounded by liver parenchyma in contrast to intrahepatic bile duct. The intrahepatic epithelium is distinct from the extrahepatic epithelium in terms of development and differentiation (Shiojiri, 1997), and the risk factors, pathogenesis, and clinical features of IHCC and EHCC are different (Strom *et al*, 1985; Nakeeb *et al*, 1996; Shaib *et al*, 2007). Although no previous studies have elucidated EGFR function in normal bile duct epithelium, EGFR overexpression might play distinct roles in IHCC and EHCC.

Vascular endothelial growth factor expression was detected frequently, being evident in about 60% of our study cases, which is consistent with previous studies (31.4–75.6%) (Hida *et al*, 1999; Tang *et al*, 2006). Our study revealed that VEGF expression was significantly associated with intrahepatic metastasis in IHCC. Vascular endothelial growth factor is a key molecule in angiogenic pathway. Angiogenesis is an essential component in the process of metastasis, and this has been partly confirmed by studies showing that microvessel density (MVD) is associated with metastasis and a poorer outcome in a range of cancers (Weidner *et al*, 1991; Zetter, 1998). It has also been reported that high MVD is an independent prognostic factor in node-negative IHCC (Shirabe *et al*, 2004) and is associated with VEGF expression in IHCC (Tang *et al*, 2006), although no study has clarified the involvement of angiogenesis in the process of metastasis in cholangiocarcinoma. Our result suggests that VEGF plays an important role in the process of cholangiocarcinoma metastasis by promoting angiogenesis.

Human epidermal growth factor receptor 2 was expressed in only 11 of 130 EHCC cases (8.5%) and in one of 106 IHCC cases (0.9%). The proportion of HER2-positive cases reported previously has varied from 4.2 to 81.8% (Ito *et al*, 2001; Aishima *et al*, 2002; Ukita *et al*, 2002; Altimari *et al*, 2003; Nakazawa *et al*, 2005), and the discrepancy may be due to differences in staining procedure or tumour location. In contrast to EGFR expression, HER2 expression was associated with more favourable clinical features, such as a polypoid macroscopic type and absence of other organ involvement. The proportion of HER2-positive cases in papillary adenocarcinoma was higher than in other histological types, consistent with some previous reports claiming that HER2 expression in cholangiocarcinoma is associated with an early disease stage (Endo *et al*, 2002; Nakazawa *et al*, 2005). Microscopically, HER2 is preferentially expressed in well differentiated component, and it is also expressed in dedifferentiated components (moderately and/or poorly differentiated components) in progressive cases. This indicates that HER2 overexpression is maintained from an early stage of carcinogenesis in cases that are HER2-positive.

Recently, the efficacy of molecular targeting therapy for various molecules including EGFR/VEGF/HER2 has been proved clinically in a wide range of cancers. Epidermal growth factor receptor inhibitor has been reported to be effective in a cholangiocarcinoma cell line (Yoon *et al*, 2004), and a phase II study of erlotinib, an EGFR inhibitor, in patients with advanced biliary cancer has been reported. In this study, the progression-free rate at 6 months as a primary end point was 17% (7/42) despite the fact that disease condition was severe, and the disease control rate was 50% (20/42) (Philip *et al*, 2006). This study suggested the clinical applicability of the EGFR inhibitor to cholangiocarcinoma. Several clinical trials demonstrating the efficacy of VEGF inhibition for other cancers have been reported (Hurwitz *et al*, 2004; Sandler *et al*, 2006), and VEGF upregulation in tumour cells is considered to be a mechanism of resistance to EGFR inhibitors (Viloria Petit *et al*, 2001). Therefore, dual inhibition of both EGFR and VEGF may exert a synergistic effect.

In summary, we have shown that EGFR and VEGF expression is relatively common in cholangiocarcinoma. Moreover, in IHCC, EGFR expression is an independent prognostic factor and VEGF expression is associated with intrahepatic metastasis. In EHCC, EGFR expression is associated with clinical factors involved in tumour progression and invasion. Our results suggest the validity and significance of molecular targeting agents for EGFR and/or VEGF pathway, and that further preclinical and clinical studies are warranted for improving the clinical outcome of cholangiocarcinoma.

## Figures and Tables

**Table 1:** Characteristics of the IHCC patients

**Factors**	**Categories**	**Population**
Age	<65 years old	54 (50.9%)
	⩾65 years old	52 (49.1%)
		
Gender	Male	64 (60.4%)
	Female	42 (39.6%)
		
Tumour size	⩽5.0 cm	55 (55.6%)
	>5.0 cm	44 (44.4%)
		
Macroscopic type	Non-mass forming	17 (16.0%)
	Mass forming	89 (84.0%)
		
Invasion of portal vein	Negative	23 (21.9%)
	Positive	82 (78.1%)
		
Invasion of hepatic vein	Negative	56 (54.9%)
	Positive	46 (45.1%)
		
Intrahepatic metastasis	Negative	75 (70.8%)
	Positive	31 (29.2%)
		
Lymph node metastasis	Negative	62 (58.5%)
	Positive	44 (41.5%)
		
UICC pT	1+2	71 (68.3%)
	3+4	33 (31.7%)
		
UICC stage	1+2	45 (42.5%)
	3A+3B+3C	61 (57.5%)
		
Histological classification	Well	22 (20.8%)
	Mod	79 (74.5%)
	Por	5 (4.7%)
		
Lymphatic vessel invasion	Negative	20 (18.9%)
	Positive	86 (81.1%)
		
Venous invasion	Negative	19 (17.9%)
	Positive	87 (82.1%)
		
Perineural invasion	Negative	29 (27.4%)
	Positive	77 (72.6%)
		
Hepatic surgical margin	Negative	89 (84.0%)
	Positive	17 (16.0%)
		
Bile duct margin	Negative	91 (85.8%)
	Positive	15 (14.2%)

Well=well differentiated adenocarcinoma; Mod=moderately differentiated adenocarcinoma; Por=poorly differentiated adenocarcinoma.

In some factors, data were not available for all cases.

**Table 2:** Characteristics of the EHCC patients

**Factors**	**Categories**	**Population**
Age	<65 years old	60 (46.2%)
	⩾65 years old	70 (53.8%)
		
Gender	Male	96 (73.8%)
	Female	34 (26.2%)
		
Tumour size	⩽3.0 cm	72 (56.3%)
	>3.0 cm	56 (43.7%)
		
Macroscopic type	Polypoid	21 (16.8%)
	Non-polypoid	104 (83.2%)
		
Depth of tumour invasion	Within FM	13 (10.0%)
	Beyond FM	117 (90.0%)
		
Invasion of portal vein	Negative	97 (74.6%)
	Positive	33 (25.4%)
		
Invasion of hepatic artery	Negative	127 (97.7%)
	Positive	3 (2.3%)
		
Lymph node metastasis	Negative	71 (54.6%)
	Positive	59 (45.4%)
		
UICC pT	1+2	49 (37.7%)
	3+4	81 (62.3%)
		
UICC stage	1A+1B	37 (28.5%)
	2A+2B+C	93 (71.5%)
		
Histological classification	Pap	20 (15.4%)
	Well	31 (23.8%)
	Mod	62 (47.7%)
	Por	17 (13.1%)
		
Lymphatic vessel invasion	Negative	16 (12.3%)
	Positive	114 (87.7%)
		
Venous invasion	Negative	19 (14.6%)
	Positive	111 (85.4%)
		
Perineural invasion	Negative	23 (17.7%)
	Positive	107 (82.3%)
		
Dissected periductal structures margin	Negative	109 (83.8%)
	Positive	21 (16.2%)
		
Bile duct margin	Negative	92 (70.8%)
	Positive	38 (29.2%)
		
Invasion to other organ	Negative	53 (40.8%)
	Positive	77 (59.2%)

FM=fibromuscular layer; Pap=papillary adenocarcinoma; Well=well differentiated adenocarcinoma; Mod=moderately differentiated adenocarcinoma; Por=poorly differentiated adenocarcinoma.

In some factors, data were not available for all cases.

**Table 3:** Multivariate analyses regarding overall survival and tumour recurrence in IHCC (Cox's proportional hazard model)

	**Overall survival**			**Tumour recurrence**		
	**HR**	**95% CI**	***P*-value**	**HR**	**95% CI**	***P*-value**
*Macroscopic type*						
Non-mass forming	1.00			1.00		
Mass forming	2.96	1.06–8.31	0.0390	3.06	1.00–9.40	0.0505
						
*Invasion of portal vein*						
Negative	1.00			1.00		
Positive	0.67	0.30–1.47	0.31	1.01	0.43–2.41	0.98
						
*Invasion of hepatic vein*						
Negative	1.00			1.00		
Positive	1.19	0.66–2.12	0.57	1.17	0.65–2.14	0.60
						
*Intrahepatic metastasis*						
Negative	1.00			1.00		
Positive	2.91	1.60–5.29	0.0005	2.36	1.31–4.25	0.0044
						
*Lymph node metastasis*						
Negative	1.00			1.00		
Positive	1.96	1.04–3.69	0.0375	2.24	1.19–4.22	0.0126
						
*Histological classification*						
Well differentiated	1.00			1.00		
Moderately differentiated	1.24	0.56–2.75	0.60	0.65	0.28–1.53	0.32
Poorly differentiated	2.09	0.58–7.49	0.26	1.35	0.32–5.72	0.69
						
*Lymphatic vessel invasion*						
Negative	1.00			1.00		
Positive	3.31	0.80–13.65	0.0982	1.37	0.41–4.56	0.61
						
*Venous invasion*						
Negative	1.00			1.00		
Positive	4.07	0.97–17.09	0.0551	6.74	1.31–34.73	0.0225
						
*Perineural invasion*						
Negative	1.00			—		
Positive	0.60	0.26–1.36	0.22	—	—	—
						
*Bile duct margin*						
Negative	1.00			—		
Positive	1.84	0.91–3.73	0.0923	—	—	—
						
*EGFR expression*						
Negative	1.00			1.00		
Positive	2.67	1.52–4.69	0.0006	1.89	1.05–3.39	0.0335

Abbreviations: CI=confidence interval; HR=hazard ratio.

**Table 4:** Multivariate analyses regarding overall survival and tumour recurrence in EHCC (Cox's proportional hazard model)

	**Overall survival**			**Tumour recurrence**		
	**HR**	**95% CI**	***P*-value**	**HR**	**95% CI**	***P*-value**
*Tumour size*						
⩽3.0 cm	1.00			—		
>3.0 cm	1.29	0.71–2.35	0.41	—	—	—
						
*Macroscopic type*						
Polypoid	1.00			—		
Non-polypoid	0.44	0.16–1.26	0.13	—	—	—
						
*Depth of tumour invasion*						
Within FM	1.00			1.00		
Beyond FM	1.26	0.19–8.60	0.81	1.16	0.24–5.57	0.85
						
*Invasion of portal vein*						
Negative	1.00			1.00		
Positive	1.48	0.81–2.69	0.20	1.59	0.92–2.75	0.94
						
*Lymph node metastasis*						
Negative	1.00			1.00		
Positive	2.03	1.16–3.55	0.0133	1.75	1.03–2.98	0.0394
						
*Histological classification*						
Papillary	1.00			1.00		
Well differentiated	3.40	0.85–13.66	0.0849	0.91	0.33–2.51	0.85
Moderately differentiated	4.23	1.08–16.50	0.0380	1.19	0.47–3.02	0.72
Poorly differentiated	13.22	3.10–56.45	0.0005	2.80	0.99–7.87	0.0516
						
*Lymphatic vessel invasion*						
Negative	1.00			1.00		
Positive	1.78	0.29–11.10	0.54	2.36	0.45–12.37	0.31
						
*Venous invasion*						
Negative	1.00			1.00		
Positive	3.93	0.81–19.12	0.0898	1.89	0.52–6.92	0.34
						
*Perineural invasion*						
Negative	1.00			1.00		
Positive	1.94	0.58–6.53	0.29	0.98	0.38–2.51	0.97
						
*Dissected periductal structures margin*						
Negative	1.00			1.00		
Positive	1.20	0.67–2.17	0.54	1.81	1.03–3.16	0.0383
						
*Invasion to other organ*						
Negative	1.00			1.00		
Positive	1.02	0.53–1.94	0.96	0.94	0.53–1.69	0.84
						
*EGFR expression*						
Negative	1.00			—		
Positive	1.04	0.55–1.96	0.90	—	—	—

HR=hazard ratio; CI=confidence interval; FM=fibromuscular layer.
