# Supplementary material for: Clinicopathological and prognostic significance of EGFR, VEGF, and HER2 expression in cholangiocarcinoma
Source: Br J Cancer. 2007 Dec 18;98(2):418–25. doi: 10.1038/sj.bjc.6604129 (PMC2361442; doi:10.1038/sj.bjc.6604129)
Supplement: Supplementary file 1 [file 6604129x1.doc]

Supplement 1 Associations between EGFR, VEGF expression and clinicopathological factors in IHCC

(Chi-squared test)

	EGFR (+)	EGFR (-)	P	VEGF(+)	VEGF(-)	P
	No.	No.		No.	No.	
Age						
≥65y.o.	15	37	0.74	28	24	0.99
<65y.o.	14	40		29	25	
Gender						
Female	10	32	0.51	25	17	0.34
Male	19	45		32	32	
Tumor size						
>5cm	13	31	0.96	25	19	0.68
≤5cm	16	39		29	26	
Macroscopic type						
Mass forming	25	64	0.70	50	39	0.26
Non-mass forming	4	13		7	10	
Invasion of portal vein						
Positive	25	57	0.21	46	36	0.28
Negative	4	19		10	13	
Invasion of hepatic vein						
Positive	14	32	0.41	26	20	0.77
Negative	13	43		30	26	
Intrahepatic metastasis						
Positive	12	19	0.0919	22	9	0.0224
Negative	17	58		35	40	
Lymph node metastasis						
Positive	14	30	0.39	21	23	0.29
Negative	15	47		36	26	
UICC pT						
1+2	18	53	0.40	35	36	0.28
3+4	11	22		20	13	
UICC stage						
1+2	10	35	0.31	25	20	0.75
3A+3B+3C	19	42		32	29	
Histological classification						
Well differentiated	4	18	0.16	11	11	0.25
Moderately differentiated	22	57		45	34	
Poorly differentiated	3	2		1	4	
Lymphatic vessel invasion						
Positive	23	63	0.77	45	41	0.54
Negative	6	14		12	8	
Venous invasion						
Positive	23	64	0.65	45	42	0.37
Negative	6	13		12	7	
Perineural invasion						
Positive	22	55	0.65	40	37	0.54
Negative	7	22		17	12	
Hepatic surgical margin						
Positive	6	11	0.42	8	9	0.54
Negative	23	66		49	40	
Bile duct margin						
Positive	5	10	0.58	8	7	0.97
Negative	24	67		49	42	

Supplement 2 Associations between EGFR, VEGF, and HER2 expression and clinicopathological factors in EHCC

(Chi-squared test)

	EGFR (+)	EGFR (-)	P	VEGF(+)	VEGF(-)	P	HER2 (+)	HER2 (-)	P
	No.	No.		No.	No.		No.	No.	
Age									
≧65y.o.	14	56	0.81	42	28	0.85	8	62	0.19
<65y.o.	11	49		35	25		3	57	
Gender									
Female	4	30	0.20	18	16	0.39	4	30	0.42
Male	21	75		59	37		7	89	
Tumor size									
>3.0cm	14	42	0.17	32	24	0.77	7	49	0.16
≦3.0cm	11	61		43	29		4	68	
Macroscopic type									
Polypoid type	0	21	0.0120	14	7	0.40	5	16	0.0078
Non-polypoid type	25	79		59	45		6	98	
Depth of tumor invasion									
Beyond FM	25	92	0.06	70	47	0.68	10	107	0.92
Within FM	0	13		7	6		1	12	
Invasion of portal vein									
Positive	6	27	0.86	17	16	0.30	1	32	0.19
Negative	19	78		60	37		10	87	
Invasion of hepatic artery									
Positive	0	3	0.39	2	1	0.79	0	3	0.59
Negative	25	102		75	52		11	116	
Lymph node metastasis									
Positive	19	40	0.0006	32	27	0.29	4	55	0.53
Negative	6	65		45	26		7	64	
UICC pT									
1+2	6	43	0.12	33	16	0.14	7	42	0.0635
3+4	19	62		44	37		4	77	
UICC stage									
1A+1B	3	34	0.0424	24	13	0.41	5	32	0.19
2A+2B+3	22	71		53	40		6	87	
Histological classification									
Papillary	2	18	0.10	10	10	0.71	5	15	0.0237
Well differentiated	3	28		19	12		3	28	
Moderately differentiated	14	48		39	23		2	60	
Poorly differentiated	6	11		9	8		1	16	
Lymphatic vessel invasion									
Positive	25	89	0.0371	69	45	0.42	11	103	0.19
Negative	0	16		8	8		0	16	
Venous invasion									
Positive	24	87	0.0946	69	42	0.10	11	100	0.15
Negative	1	18		8	11		0	19	
Perineural invasion									
Positive	24	83	0.0459	65	42	0.45	9	98	0.96
Negative	1	22		12	11		2	21	
Dissected periductal structures margin									
Positive	5	16	0.56	11	10	0.49	1	20	0.51
Negative	20	89		66	43		10	99	
Bile duct margin									
Positive	7	31	0.88	24	14	0.56	5	33	0.22
Negative	18	74		53	39		6	86	
Invasion to other organ									
Positive	17	60	0.32	42	35	0.19	3	74	0.0242
Negative	8	45		35	18		8	45	

FM, fibromuscular layer
